# Robustness of nutrient-based NOVA food classification under profile-separated nested validation

**DOI:** 10.3389/fnut.2026.1916576

**Published:** 2026-09-17

**Authors:** Sıla Sinem Gören, Raja Hashim Ali, Talha Ali Khan, Iftikhar Ahmed

**Affiliations:** 1 Department of Business, University of Europe for Applied Sciences, Potsdam, Brandenburg, Germany; 2 Department of Artificial Intelligence, Ghulam Ishaq Khan Institute of Engineering Sciences and Technology, Topi, Pakistan

**Keywords:** class imbalance, nested cross-validation, NOVA classification, nutrient profiling, ultra-processed foods

## Abstract

**Introduction:**

Nutrient profiles contain information associated with food processing, but estimates of automated NOVA classification can be distorted by ambiguous record linkage, severe class imbalance, duplicate profiles, and non-nested model selection. This study evaluates how well four-class NOVA labels can be recovered from nutrient composition after addressing these sources of optimism.

**Methods:**

The analysis used 2,484 manually labeled foods from the USDA Food and Nutrient Database for Dietary Studies, represented by 99 nutrient variables. Identical nutrient profiles were assigned to the same fold, and logistic regression, a radial-basis support vector machine, random forest, and histogram gradient boosting were compared with a prior-probability baseline using three repeats of five-fold nested stratified group cross-validation. Hyperparameters and all transformations were selected within each outer training partition. Performance was summarized using class-balanced and chance-corrected metrics, corrected repeated-cross-validation tests, and fold-stable multiclass Shapley attribution.

**Results:**

Histogram gradient boosting achieved accuracy 0.921 and macro-F1 0.814 (SD 0.029; 95% fold interval 0.798–0.831). Random forest reached macro-F1 0.788; the mean difference of 0.026 was not statistically reliable after repeated-fold correction (Holm-adjusted *p* = 0.203). Class-specific F1 was 0.639 for NOVA 2 and 0.958 for NOVA 4.

**Discussion:**

Nutrient measurements therefore provide a strong but incomplete correlate of NOVA class. The models are suitable for methodological screening within this data source, but they do not replace ingredient- and process-based expert classification; external validation on contemporary branded foods and independently adjudicated labels remains necessary.

## Highlights

- Direct food-code alignment yielded 2,484 manually NOVA-labeled foods.
- Identical nutrient profiles were kept together in every validation split.
- Repeated nested validation compared four classifiers with a prior baseline.
- Boosting reached 0.814 macro-F1; random forest was not significantly worse.
- Shapley rankings were reported with across-fold stability, not one fitted model.

## 1 Introduction

The NOVA system groups foods according to the nature, extent, and purpose of industrial processing: unprocessed or minimally processed foods (NOVA 1), processed culinary ingredients (NOVA 2), processed foods (NOVA 3), and ultra-processed foods (NOVA 4) (1, 2). It is used in nutritional epidemiology and policy research, but classification can require detailed ingredient and manufacturing information that is not consistently available. The boundaries between classes can also be difficult to apply consistently, especially for composite foods (3, 4).

Nutrient-based machine learning offers a complementary route. Processing and formulation can alter concentrations of sodium, water, carbohydrates, fats, micronutrients, and other constituents, creating multivariate patterns that are detectable even when ingredient lists are unavailable. Menichetti et al. (5) developed FoodProX from Food and Nutrient Database for Dietary Studies (FNDDS) nutrient profiles and demonstrated high discrimination of manually assigned NOVA classes. Related work has used nutrient-density and formulation markers to characterize ultra-processed foods (6, 7), while machine-learning applications in nutrition extend to consumption modeling and dietary-pattern analysis (8). These studies establish that structured food and consumption data can support useful prediction, but they also make clear that predictive association is not equivalent to a processing definition or causal mechanism.

Reliable evaluation is particularly important here. NOVA 4 commonly dominates available labeled samples, so accuracy and weighted measures can conceal poor performance for NOVA 2. Hyperparameter selection and preprocessing performed before evaluation can produce optimistic estimates, and identical nutrient vectors placed in both training and test sets can create direct feature-profile leakage. A single random partition also provides no estimate of split-to-split variability. Nested cross-validation separates model selection from assessment (9); paired corrected tests are needed because repeated cross-validation scores are dependent (10).

An exploratory analysis associated with this study linked 8,790 USDA Standard Reference records to NOVA labels by normalized food names and retained 496 items. Audit of that process showed that most retained records relied on the first two words of the description rather than an exact identifier. The resulting sample was therefore unsuitable as the primary confirmatory dataset. The present study instead uses the directly aligned, manually labeled FNDDS records and treats the exploratory analysis only as a traceability check.

This reanalysis addresses three questions:

1. How accurately can NOVA class be predicted from nutrient measurements when identical profiles are separated at the group level and model selection is nested?
2. How do linear, kernel, bagging, and boosting classifiers compare under class-balanced and chance-corrected metrics, particularly for NOVA 2?
3. Which nutrients contribute most strongly to predictions, and how stable are their Shapley rankings across held-out folds?

The contribution is a reproducible robustness benchmark, not a claim that nutrients redefine NOVA or that an algorithm supersedes expert assessment.

## 2 Materials and methods

### 2.1 Data provenance and analytical sample

The primary sample was reconstructed from the source-data workbook accompanying Menichetti et al. (5). The workbook links FNDDS 2009–2010 food codes to 99 natural-log nutrient variables and contains a separate set of manual NOVA assignments. Food codes, rather than food-name similarity, were used for alignment. One standalone salt record appears in the food-description sheet but not in the nutrient matrix; after accounting for this documented offset, the remaining 7,253 food codes and nutrient rows aligned exactly. Joining the 2,484 manually classified food codes to this matrix produced 2,484 analytical records with no missing nutrient values and no duplicate food codes (Figure 1).

**Figure 1:**
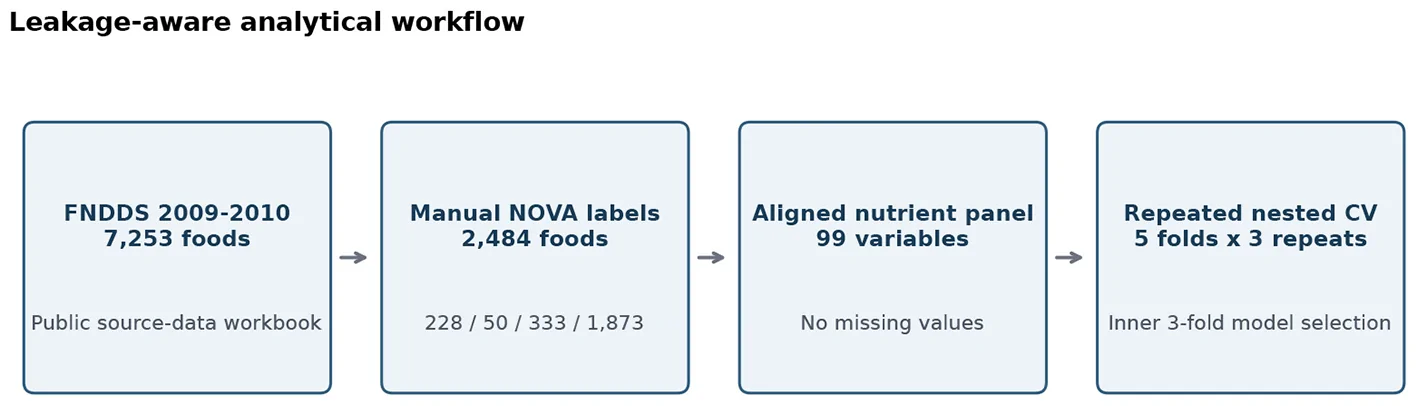
Data provenance and evaluation workflow. The exploratory 496-record name match is reported only for traceability. The primary analysis uses direct food-code alignment of 2,484 manually labeled FNDDS foods. Identical 99-variable nutrient profiles are grouped before nested validation.

The source matrix encodes structural zeros as −20 and otherwise supplies natural-log nutrient values. These values were retained as provided; no additional logarithmic transformation or imputation was applied. This avoids reconstructing concentrations from a transformed research dataset and then transforming them again. The 99 variables include macronutrients, minerals, vitamins, fatty acids, and related food-composition measures.

The class distribution was 228 NOVA 1 foods (9.18%), 50 NOVA 2 foods (2.01%), 333 NOVA 3 foods (13.41%), and 1,873 NOVA 4 foods (75.40%) (Figure 2). There were 2,160 unique nutrient profiles. A total of 591 records belonged to 267 repeated-profile groups; eight groups (17 records) contained more than one NOVA label despite having identical nutrient vectors. Those conflicts demonstrate that nutrient composition alone cannot uniquely determine NOVA class.

**Figure 2:**
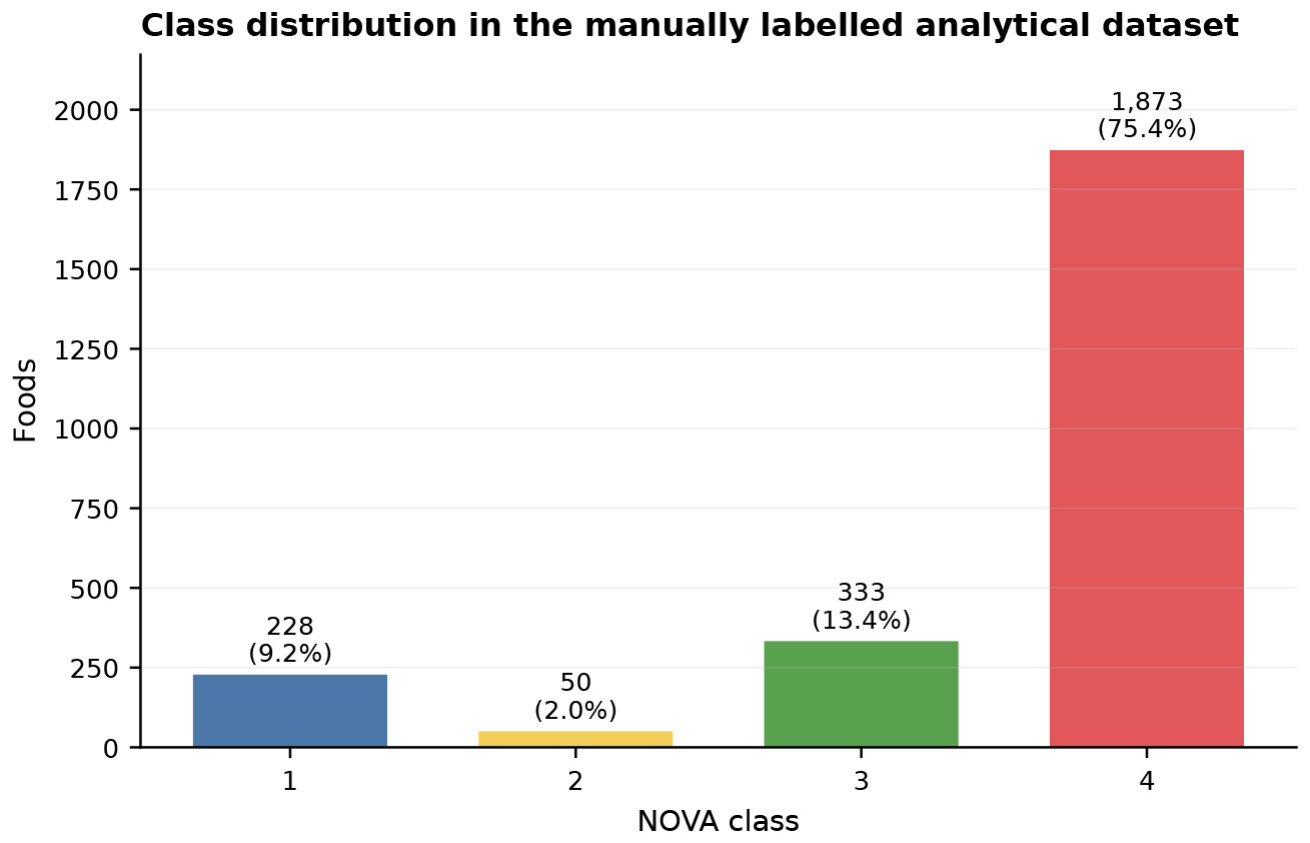
Class distribution in the directly aligned analytical sample (*n* = 2,484). Counts and proportions are shown because overall accuracy is strongly influenced by the NOVA 4 majority.

Table 1 reconciles every sample size reported in the revision. The 496-record exploratory sample is not combined with the FNDDS sample and does not contribute to primary model estimates.

**Table 1:** Reconciliation of source, analytical, validation-group, and exploratory sample sizes.

Stage	Records/groups	Role in the revised study
FNDDS nutrient matrix	7,253	Source foods with 99 aligned nutrient variables
Manual NOVA label set	2,484	Food-code labels available for direct alignment
Primary analytical sample	2,484	No missing values; 99 nutrient predictors
Unique nutrient profiles	2,160	Exact 99-variable profiles used as validation groups
Repeated-profile groups	267	591 records kept within folds
Conflicting-label profiles	8	17 records share predictors but not labels
Exploratory Standard Reference file	8,790	Used only to audit the former name-linkage workflow
Exploratory matched sample	496	56 exact and 440 first-two-token matches
Exploratory test partition	100	Single 80/20 split; not used for primary inference

### 2.2 Audit of the exploratory record linkage

For traceability, the original 8,790-record Standard Reference file from USDA FoodData Central (16) was processed according to the associated computational workflow. Food descriptions were lower-cased, punctuation was removed, and records were joined first by the complete normalized name and then by the first two normalized tokens. Duplicate Standard Reference identifiers were retained only once. This produced 496 records: 56 exact-name matches and 440 first-two-token matches. Among retained records, 37 had more than one candidate NOVA label and one record had 27 possible source rows. In addition, imputation, log transformation, and standardization had been fitted before the single train-test split. The reproduced random-forest result from that workflow was 0.880 accuracy and 0.796 macro-F1 on 100 test records. These values are reported as a reproducibility audit, not as evidence of generalization.

### 2.3 Profile-separated nested validation

Exact duplicate profiles were assigned a common group identifier derived from all 99 nutrient values. The outer evaluation used three repetitions of five-fold stratified group cross-validation (15 held-out folds). Each profile group appeared in the test set once per repetition and never appeared in both training and test data within a fold. Within every outer training partition, a separate three-fold stratified group cross-validation selected hyperparameters by macro-F1. Thus, the outer test observations were not used for preprocessing, model choice, hyperparameter selection, or explanation-background construction.

The repeated scheme was chosen to expose split variability while preserving the minority class as far as group constraints allowed. The unit of evaluation remained the food record, while grouping prevented identical predictors from crossing the assessment boundary. A first repetition of mutually exclusive out-of-fold predictions was used for the confusion matrix and class-specific precision, recall, and F1 so that each food contributed once to these descriptive summaries.

### 2.4 Models, preprocessing, and class imbalance

Five specifications were compared: a prior-probability dummy classifier, multinomial logistic regression, radial-basis-function support vector machine (RBF-SVM), random forest (11), and histogram gradient boosting (12). Logistic regression and RBF-SVM were standardized using means and standard deviations fitted within each inner training fold. Tree models used the supplied log nutrient values directly. The learned models used class-balanced loss weights; random forest used balanced subsample weights. Oversampling was not used because synthetic interpolation among nutrient profiles could create foods with no meaningful compositional interpretation.

The complete search spaces are shown in Table 2. Grid search was exhaustive within these pre-specified ranges. Because nested validation produces a separately optimized model in each outer fold, there is no single “final” parameter vector underlying the performance estimate. Fold-specific selections are supplied in machine-readable form. A separate five-fold group-stratified selection on all 2,484 records defines a reproducible full-data refit specification, but its internal selection score is not reported as test performance.

**Table 2:** Models, preprocessing, inner search spaces, and full-data refit specifications.

Model	Fold-local preprocessing/imbalance	Inner search space	Full-data refit setting
Prior baseline	Prior probabilities; no weighting	None	No fitted hyperparameters
Logistic regression	Standardized; class balanced	*C*∈{0.1, 1, 10}	*C* = 10
RBF-SVM	Standardized; class balanced	*C*∈{1, 5, 10}; γ∈{scale, 0.01}	*C* = 5; γ = scale
Random forest	Unscaled; balanced subsamples	300 trees; depth ∈{None, 16}; minimum leaf ∈{1, 3, 6}	300 trees; no depth limit; minimum leaf 1
Histogram gradient boosting	Unscaled; class balanced	Learning rate ∈{0.05, 0.10}; leaves ∈{15, 31}; minimum leaf ∈{10, 25}	Learning rate 0.10; 31 leaves; minimum leaf 25

Performance is estimated only by the outer folds.

### 2.5 Performance measures and statistical comparison

Macro-F1 was the primary measure because it gives equal weight to all four classes. We also report accuracy, macro precision, macro recall, weighted F1, balanced accuracy, Cohen's κ, Matthews correlation coefficient (MCC), and one-vs.-rest macro- and prevalence-weighted area under the receiver-operating-characteristic curve (AUC). Precision, recall, and F1 are explicitly macro-averaged unless described as class-specific or weighted.

For each model and metric, the 15 outer-fold values are summarized by the mean, sample standard deviation, and a two-sided 95% *t* interval over folds. These intervals describe variation in the repeated validation experiment; they are not external-population confidence intervals. Pairwise macro-F1 differences between the best-performing model and each alternative were evaluated with the corrected repeated-cross-validation *t* statistic (10). Holm's method adjusted the resulting *p*-values across four comparisons. Statistical evidence was interpreted together with effect size and class-specific performance rather than through a binary significance threshold alone.

### 2.6 Multiclass Shapley analysis and stability

Feature attribution used a model-agnostic Monte Carlo permutation implementation of interventional Shapley values (13, 14). For each of the five outer models from the first repetition, up to 25 held-out observations per NOVA class were sampled. Each observation was paired with six independently sampled training-background records, and feature-entry order was randomized once per background. Contributions were computed for all four predicted class probabilities. Global importance was defined as the mean absolute Shapley contribution across evaluated observations and the four class outputs, normalized to sum to one within each fold.

Stability was evaluated by the mean and standard deviation of importance, mean rank, top-10 frequency, and pairwise Spearman rank correlation across the five outer folds. This design avoids deriving an explanation from the same observations used to fit the corresponding model. Class-specific contribution plots show signed effects for the NOVA 4 probability; they describe the fitted decision function and do not establish causal or biological mechanisms.

### 2.7 Software and reproducibility

The complete analysis was executed in Python 3.12.13 using pandas 2.2.3, NumPy 2.3.5, SciPy 1.17.0, scikit-learn 1.8.0 (15), and Matplotlib 3.10.8. A fixed seed (20,260,803) controls split generation, estimator initialization, and Shapley sampling. The analysis script, checksums, fold assignments, predictions, selected hyperparameters, performance tables, attribution outputs, and figure-generation code accompany the revision.

## 3 Results

### 3.1 Primary model comparison

Histogram gradient boosting produced the highest mean macro-F1 (0.814; SD 0.029; 95% fold interval 0.798–0.831), with accuracy 0.921, balanced accuracy 0.810, and macro AUC 0.980. Random forest was the closest alternative (macro-F1 0.788). RBF-SVM and logistic regression reached macro-F1 0.723 and 0.611, respectively. The prior baseline attained accuracy 0.754 because NOVA 4 dominated the sample, but its macro-F1 was only 0.215. Figure 3 shows the primary and selected secondary metrics with across-fold standard deviations; the numerical results and 95% fold intervals appear in Table 3.

**Figure 3:**
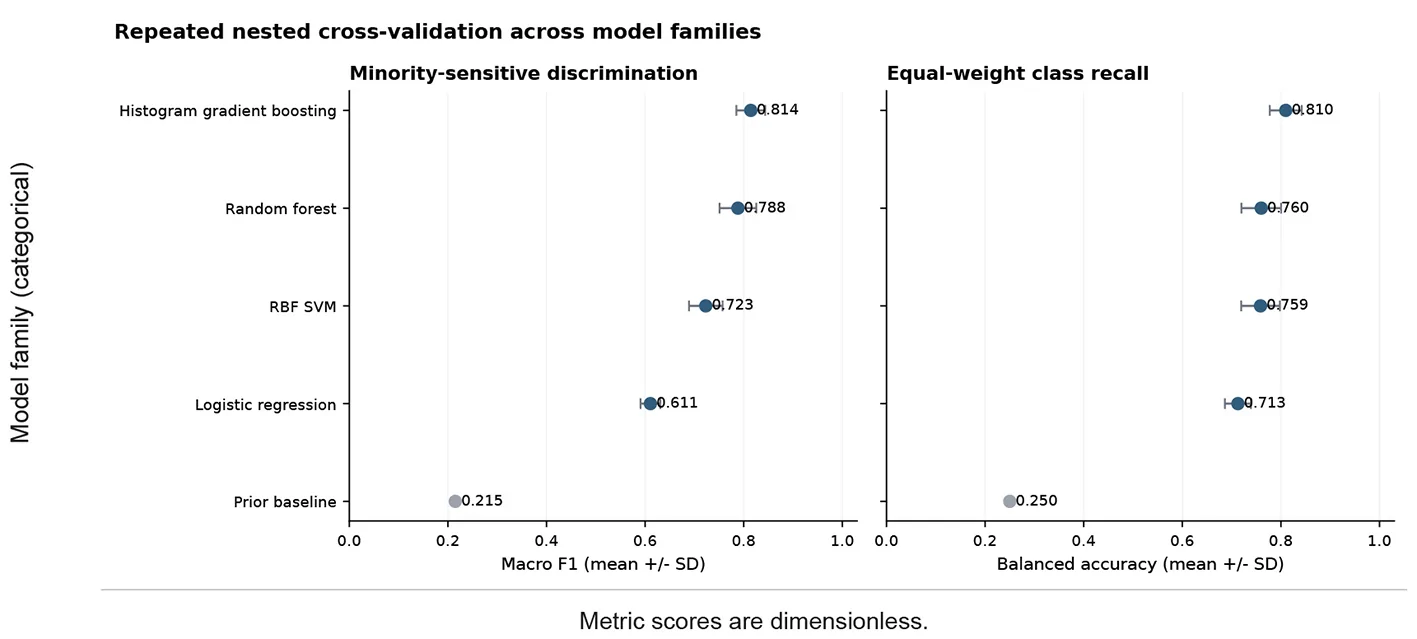
Profile-separated repeated nested-validation performance. Points are means across 15 outer folds and error bars are one standard deviation. Macro-F1 balances precision and recall across classes, while balanced accuracy is the mean class recall.

**Table 3:** Repeated profile-separated nested-validation performance.

Model	Accuracy	Macro-F1	95% fold interval	Weighted F1	Balanced acc.	κ	MCC	Macro AUC
Prior baseline	0.754 ± 0.001	0.215 ± 0.000	[0.215, 0.215]	0.648	0.250	0.000	0.000	0.500
Logistic regression	0.788 ± 0.014	0.611 ± 0.020	[0.600, 0.622]	0.808	0.713	0.560	0.577	0.921
RBF-SVM	0.869 ± 0.011	0.723 ± 0.034	[0.704, 0.742]	0.875	0.759	0.697	0.700	0.960
Random forest	0.908 ± 0.012	0.788 ± 0.037	[0.768, 0.809]	0.907	0.760	0.768	0.770	0.976
Histogram gradient boosting	0.921 ± 0.014	0.814 ± 0.029	[0.798, 0.831]	0.921	0.810	0.806	0.807	0.980

Values are means across 15 outer folds; standard deviations are shown for accuracy and the primary macro-F1 measure.

### 3.2 Class-specific errors

For histogram gradient boosting, NOVA 4 had precision 0.958, recall 0.957, and F1 0.958. NOVA 2 remained the most difficult class, with precision 0.660, recall 0.620, and F1 0.639 from 50 foods. NOVA 1 and NOVA 3 achieved F1 0.844 and 0.802 (Table 4). The out-of-fold confusion matrix (Figure 4) shows that errors were concentrated in the minority and adjacent processing categories rather than the NOVA 4 majority. Identical nutrient profiles with conflicting manual labels impose an irreducible ceiling on any deterministic nutrient-only classifier.

**Table 4:** Class-specific out-of-fold performance of histogram gradient boosting in the first repetition.

Class	Description	Support	Precision	Recall	F1
NOVA 1	Unprocessed or minimally processed	228	0.856	0.833	0.844
NOVA 2	Processed culinary ingredients	50	0.660	0.620	0.639
NOVA 3	Processed foods	333	0.790	0.814	0.802
NOVA 4	Ultra-processed foods	1,873	0.958	0.957	0.958

Each food contributes once.

**Figure 4:**
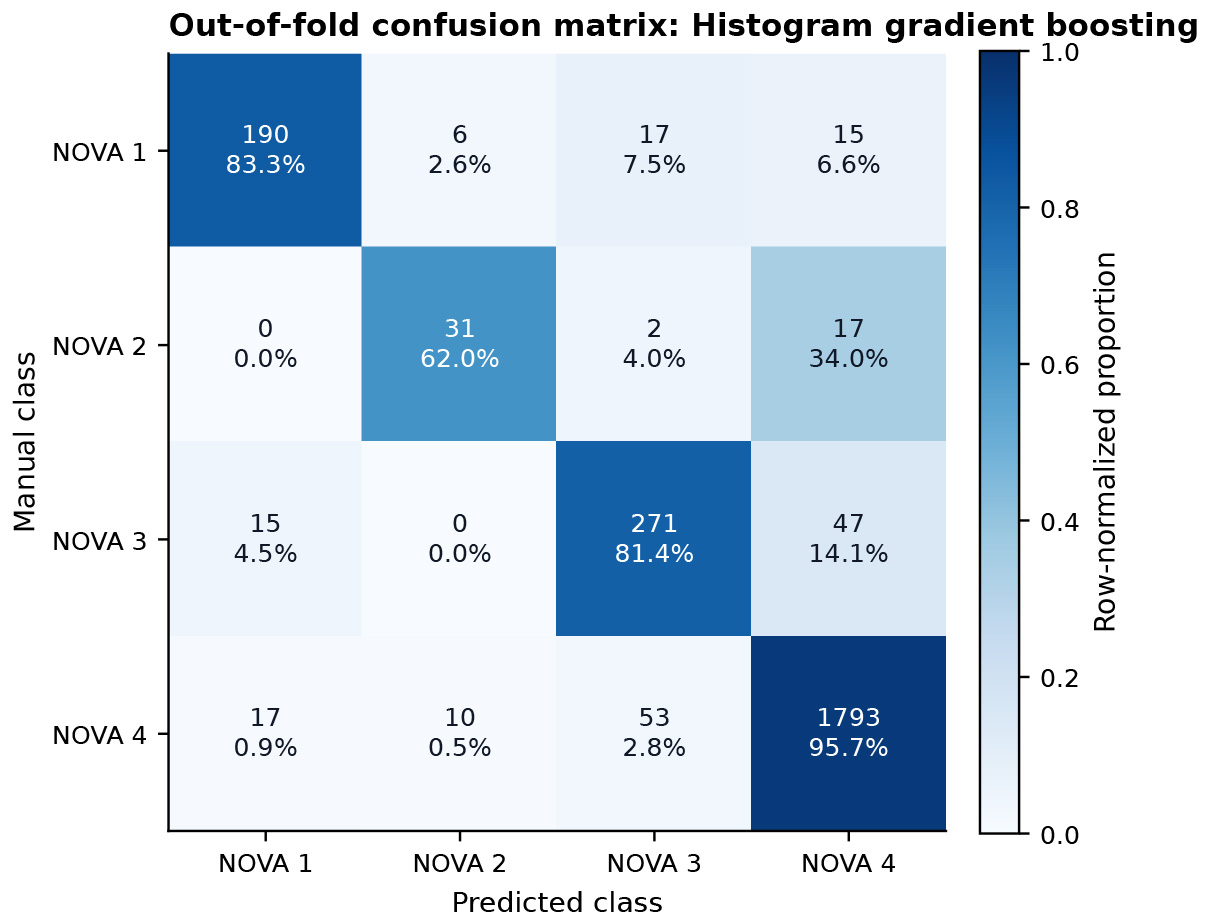
Out-of-fold confusion matrix for the best model in the first five-fold repetition. Each of the 2,484 foods contributes once. Values are row-normalized percentages, with counts annotated.

### 3.3 Paired model comparisons and selected hyperparameters

Histogram gradient boosting exceeded random forest by mean macro-F1 0.026, but this difference was not statistically reliable (corrected *t*_(14)_ = 1.34, Holm-adjusted *p* = 0.203). Its advantages over RBF-SVM, logistic regression, and the prior baseline remained significant after Holm adjustment. The corrected comparisons are summarized in Table 5. The comparisons account for reuse of records across repeated folds and are adjusted for the four pairwise tests. They do not address external-dataset transportability.

**Table 5:** Corrected repeated-cross-validation comparisons against histogram gradient boosting.

Comparison model	Mean Δ macro-F1	Corrected *t*	df	Raw *p*	Holm *p*
Prior baseline	0.599	36.52	14	< 0.001	< 0.001
Logistic regression	0.204	13.82	14	< 0.001	< 0.001
RBF-SVM	0.091	5.16	14	< 0.001	< 0.001
Random forest	0.026	1.34	14	0.203	0.203

Positive differences favor gradient boosting.

For the full-data refit, the selected specifications were logistic regression *C* = 10; RBF-SVM *C* = 5 with γ = scale; random forest with 300 trees, unrestricted depth, and minimum leaf size 1; and histogram gradient boosting with learning rate 0.10, 31 maximum leaves, and minimum leaf size 25. These settings are provided to reproduce a model fitted on all available records; only the nested outer-fold estimates in Table 3 quantify predictive performance.

### 3.4 Feature contributions and stability

Sodium had the highest normalized mean absolute contribution (0.113) and ranked first in all five fold. Water, total sugars, carbohydrate, and potassium completed the five highest mean importances. Sodium, water, total sugars, carbohydrate, potassium, and folic acid appeared in the top 10 in every fold. The leading features and across-fold stability statistics are reported in Table 6. Pairwise feature-rank correlations ranged from 0.912 to 0.955, with median 0.945. Figure 5 displays both importance magnitude and rank stability. Figure 6 shows the direction of feature contributions to the NOVA 4 probability. The contribution patterns are multivariate: no single nutrient is sufficient to define processing level.

**Table 6:** Top global approximate Shapley features and across-fold stability.

Feature	Mean importance	SD	Mean rank	Top-10 folds
Sodium	0.113	0.013	1.0	5/5
Water	0.076	0.005	2.0	5/5
Sugars, total	0.055	0.006	3.8	5/5
Carbohydrate	0.053	0.012	3.8	5/5
Potassium	0.045	0.006	5.2	5/5
Folic acid	0.040	0.011	6.4	5/5
Thiamin	0.035	0.007	7.8	4/5
Fiber, total dietary	0.029	0.002	9.2	4/5
Iron	0.029	0.004	10.2	2/5
Selenium	0.026	0.004	11.8	2/5

Importance is normalized within each fold.

**Figure 5:**
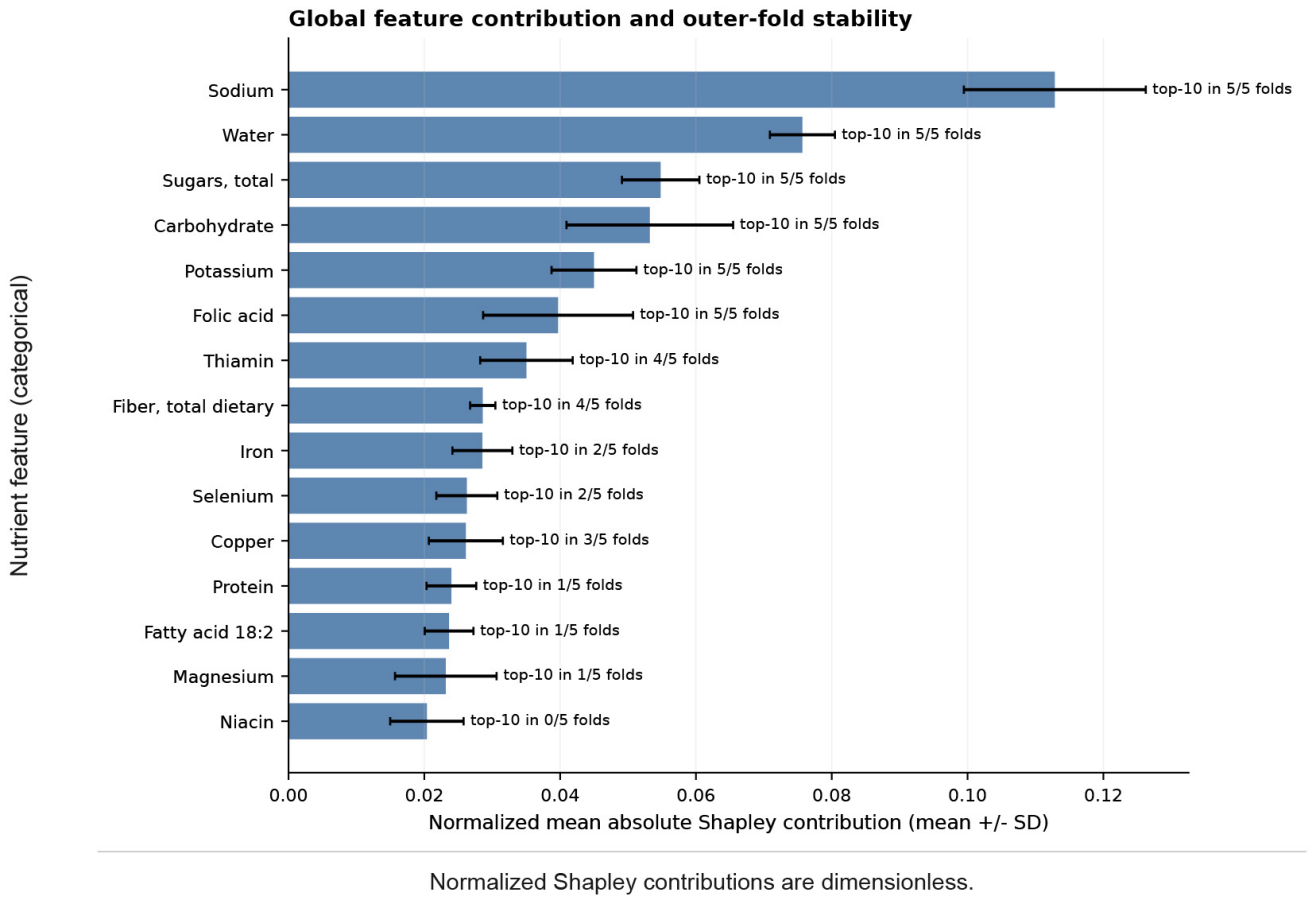
Global approximate Shapley importance and stability across five held-out folds. Bars show mean normalized absolute contribution; error bars show one standard deviation. The accompanying rank information prevents a single fitted model from being treated as a stable global explanation.

**Figure 6:**
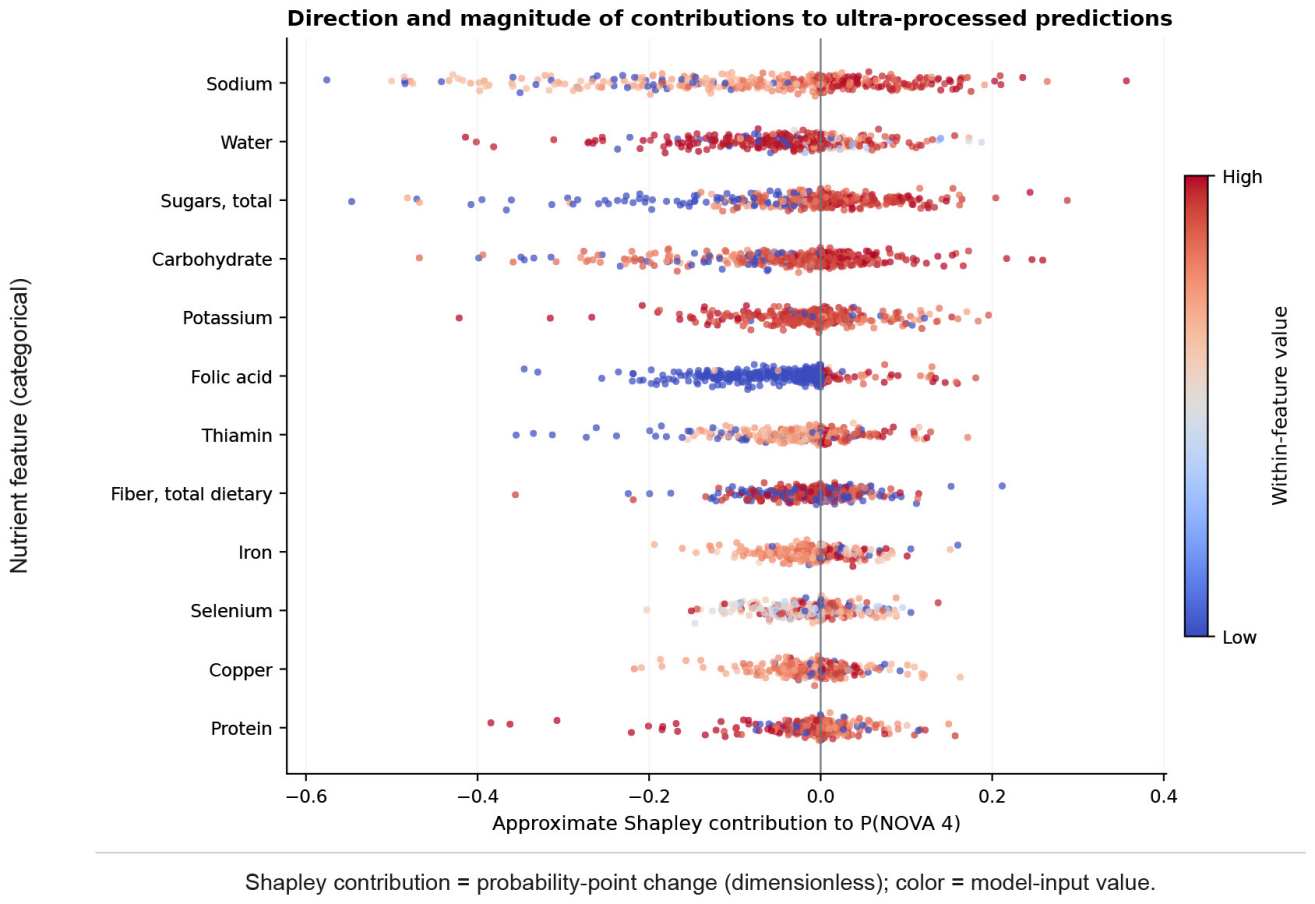
Approximate Shapley contributions to the predicted NOVA 4 probability for held-out foods. Color represents the within-feature value after the model's preprocessing. Positive values increase and negative values decrease the predicted NOVA 4 probability for the fitted fold model.

## 4 Discussion

This study found that nutrient profiles carry substantial information about manually assigned NOVA classes, but the strength of that evidence depends on data provenance and validation design. The direct food-code-aligned sample is five times larger than the exploratory name-matched sample and contains a less extreme, although still severe, representation problem for NOVA 2. Keeping repeated nutrient profiles together prevents a classifier from being tested on a feature vector it has already encountered. Under this stricter design, histogram gradient boosting reached macro-F1 0.814 and balanced accuracy 0.810, while random forest reached macro-F1 0.788. The resulting performance is strong enough to support screening, but not perfect or uniform across classes.

The result is consistent with the premise that processing and formulation alter combinations of nutrients (5). It does not imply that nutrients are equivalent to the NOVA definition. NOVA considers the purpose and extent of processing, ingredient formulation, and use of industrial substances (2); two foods can therefore share a nutrient profile yet receive different labels. The eight conflicting repeated-profile groups observed here provide direct empirical evidence of that non-identifiability. Ingredient lists, additives, preparation methods, and expert adjudication remain relevant inputs.

Class imbalance materially affects interpretation. NOVA 4 represents three quarters of the sample, allowing all learned models to attain comparatively high accuracy and weighted F1. Macro-F1, balanced accuracy, and the class-specific table reveal the more difficult NOVA 2 problem. Class weighting improved the role of minority observations during fitting, but it cannot replace missing diversity. Resampling was deliberately avoided because synthetic nutrient vectors would not necessarily correspond to real foods. Future data collection should prioritize additional independently labeled NOVA 2 and borderline NOVA 3 records.

The model comparison also tempers claims of algorithmic superiority. Histogram gradient boosting had the highest average macro-F1, yet its 0.026 advantage over random forest did not survive the dependence-corrected comparison (*p*_Holm_ = 0.203). By contrast, its larger differences from linear and kernel baselines were statistically reliable. Statistical comparison is useful, but a difference among models trained and tested within one source cannot establish general superiority. Transport to newer FNDDS releases, branded-food databases, other countries, and independently adjudicated labels is the more consequential test.

The Shapley analysis identifies nutrients that the fitted models use, not biomarkers of industrial processing. Sodium was consistently ranked first, followed on average by water, total sugars, carbohydrate, and potassium. The median pairwise rank correlation of 0.945 indicates substantial stability for the leading pattern, while lower-ranked features varied across folds. Correlated nutrient measures can divide or exchange attribution, and rankings depend on the background distribution. Reporting across-fold stability and signed class-specific contributions provides a more defensible explanation than a single impurity-importance plot, but it does not support causal inference.

The exploratory 496-record workflow remains informative as a cautionary reproducibility example. Its single split yields apparently strong performance, but 440 of 496 records were obtained by a first-two-token match, 37 retained foods had multiple candidate labels, and preprocessing preceded the split. The old sample sizes of approximately 12,000 and 3,600 test observations could not be reconciled with executable data and have been removed. The revised analysis reports one auditable path from 7,253 source foods to 2,484 labeled analytical records and 15 held-out folds.

### 4.1 Strengths and limitations

Strengths include direct identifier-based label alignment, explicit reconciliation of all sample counts, profile-separated repeated nested validation, fold-local model selection, multiple imbalance-sensitive metrics, a chance baseline, corrected paired comparisons, and fold-level explanation stability. Every numerical result is generated from machine-readable fold outputs.

Several limitations remain. First, this is a secondary analysis of one historical US food-composition source and shares its labels and nutrient measurements with the FoodProX study (5); it is a robustness benchmark, not an independent external replication. Second, no ingredient lists, additive indicators, preparation metadata, or manufacturing-process variables were available. Third, manual NOVA labels can be uncertain and no new multi-rater adjudication was conducted. Fourth, repeated cross-validation intervals quantify split variability within the source data and should not be interpreted as population uncertainty. Fifth, the class distribution is highly imbalanced, especially for NOVA 2. Sixth, Monte Carlo Shapley values are approximations and correlated predictors complicate attribution. Finally, grouping exact duplicate profiles does not address near-duplicate foods or broader product-family dependence.

### 4.2 Implications and future work

Nutrient-based classification may be useful for screening records that warrant expert review, prioritizing missing labels, or comparing modeling protocols. It should not be used as an autonomous regulatory label or as evidence that a food's processing history can be recovered perfectly from nutrient composition. A prospective validation study should pre-register the label protocol, obtain independent ratings and adjudication, preserve product-family groups, and evaluate temporally or geographically external data. Hybrid models combining nutrients with ingredients and processing metadata should be compared against expert agreement, with calibration and abstention rules for ambiguous foods.

## 5 Conclusion

Direct alignment of 2,484 manually labeled foods and profile-separated nested validation provide a coherent estimate of nutrient-based NOVA classification. Histogram gradient boosting achieved mean macro-F1 0.814 and accuracy 0.921, while random forest reached macro-F1 0.788; their difference was not statistically reliable after corrected repeated-fold testing. The remaining errors are not merely computational: identical nutrient profiles can carry different NOVA labels, and the minority NOVA 2 class remains comparatively difficult. Nutrient profiles should therefore be interpreted as correlates of processing-related formulation, not as a replacement definition for NOVA. The practical value of these models is as transparent decision support within a clearly bounded data domain. Claims of expert replacement, causal nutrient markers, or large-scale deployment require independent labels, ingredient and process information, and external prospective validation.

## Data Availability

The original contributions presented in the study are included in the article/supplementary material, further inquiries can be directed to the corresponding author.
